# Development and Evaluation of ^18^F-IRS for Molecular Imaging Mutant EGF Receptors in NSCLC

**DOI:** 10.1038/s41598-017-01443-7

**Published:** 2017-06-09

**Authors:** Yan Song, Zunyu Xiao, Kai Wang, Xiance Wang, Chongqing Zhang, Fang Fang, Xilin Sun, Baozhong Shen

**Affiliations:** 1 Molecular Imaging Research Center, Harbin Medical University, Harbin, Heilongjiang China; 2 TOF-PET/CT/MR center, The Fourth Hospital of Harbin Medical University, Harbin, Heilongjiang China; 3 Molecular Imaging Program at Stanford (MIPS), Department of Radiology, Stanford University School of Medicine, Stanford, California USA

**Keywords:** Cancer imaging, Non-small-cell lung cancer

## Abstract

To prepare and evaluate a new radiotracer ^18^F-IRS for molecular imaging mutant EGF Receptors *in vitro* and *vivo*. Uptake and efflux of ^18^F-IRS were performed with four NSCLC cell lines including HCC827, H1975, H358 and H520. *In vivo* tumor targeting and pharmacokinetics of the radiotracers were also evaluated in HCC827, H1975, H358 and H520 tumor-bearing nude mice by PET/CT imaging. *Ex vivo* biodistribution assays were performed to quantify the accumulation of ^18^F-IRS *in vivo*. We also performed ^18^F-IRS PET/CT imaging of three patients with NSCLC. We labeled this small molecule with QD620 for flow cytometry and confocal imaging analyses. The uptakes of ^18^F-IRS by HCC827 and HCC827 tumors were significantly higher than those of H358, H1975 and H520, and they were reduced by the addition of 100 μM of gefitinib. Biodistribution experiments showed an accumulation of ^18^F-IRS in tumors of HCC827 xenografts. Flow cytometry and confocal imaging with QD620-IRS further demonstrated that binding specifically to HCC827 cells. ^18^F-IRS accumulation was preferential in the tumor, which was NSCLC with responsive EGFR exon 19 deleted. ^18^F-IRS showed high binding stability and specificity to 19 exon deleted EGFR mutation *in vitro* and *vivo*.

## Introduction

Despite decades of research and prevention strategies, lung cancer remains the most common cancer worldwide, with approximately 1.35 million new cases annually^1^. Lung cancer has the highest cancer mortality rate, accounting for almost 30% of cancer-related deaths^2^. Non-small cell lung cancer (NSCLC) is the most prevalent type of lung cancer, comprising 85% of all lung cancers. Notably, 15–30% of NSCLC patients have tumors harboring activating mutations in the epidermal growth factor receptor (EGFR) that have been associated with a response to EGFR tyrosine kinase inhibitors (TKIs)^1, 3^, and the most frequently detected alterations were small deletions in exon 19 (35–45%) that eliminate amino acids 747–750 (Leu-Arg-Glu-Ala), located around the active site of the kinase^4^.

EGFR is an oncogene that plays a prominent role in tumor growth and metastasis by acting in concert with other ERBB (human epidermal growth factor receptor) family members, such as ERBB2 and ERBB3^5–8^. EGFR is expressed in many human epithelial malignancies including NSCLC^6, 9^. Upon ligand binding, EGFRs homodimerize or heterodimerize thereby leading to autophosphorylation and subsequent activation of downstream mitogen-activated protein kinase (MAPK) and phosphatidylinositol-3-kinase (PI3K)/AKT signaling cascades, which regulate cell proliferation, angiogenesis, invasion, and metastasis^10, 11^. In recent years, many EGFR-targeted agents have been synthesized to inhibit the tyrosine kinase domain of EGF^12, 13^. One of the most widely used EGFR-TKIs is gefitinib (Iressa, ZD1839), a fluorine-containing aniline-quinazoline that disrupts EGFR kinase activity by binding to the intracellular tyrosine kinase domain of EGFR^14^. Although most NSCLC patients with tumors bearing EGFR mutations show high response and survival rates when treated with EGFR-TKIs, the vast majority of these patients eventually develop resistance to the treatment. A T790M secondary mutation has been identified as the most frequent mechanism for acquired resistance in such patients^15, 16^. EGFR-activating mutations and the T790M secondary mutation are therefore useful biomarkers for identifying patients who will benefit from or became resistant to therapy with EGFR-TKIs^17^. Thus, there is currently a great need for new approaches that could better characterize the mutation status and expression levels of EGFR in NSCLC.

Positron emission tomography (PET) is one of the most powerful noninvasive imaging technologies for the production of high-resolution, three-dimensional images within deep tissue that is currently used in clinical applications. PET imaging can identify pre-symptomatic biochemical changes in body tissues where no evidence of abnormality is detected using computed tomography (CT) or magnetic resonance imaging (MRI), or before structural changes occur from disease^18, 19^.

Here, we report the synthesis of a novel radiotracer, ^18^F-N-(3-chloro-4-fluorophenyl)-7-(2(2- (2-(2-(4-fluorine)ethoxy)ethoxy)-ethoxy)-6-(3-morpholinopropoxy)quinazolin-4-amine (^18^F-IRS) for PET imaging that was selective and reversible binding to 19 exon deleted EGFR mutation. We have also characterized the *in vitro* and *in vivo* behavior of ^18^F-IRS in cultured cells and NSCLC xenografted mouse models, respectively. To verify ^18^F-IRS could be used to noninvasively detect EGFR expression in patients with NSCLC, we performed ^18^F-IRS PET/CT imaging of three patients with NSCLC.

## Results

### Molecular Docking

**Figure 1:**
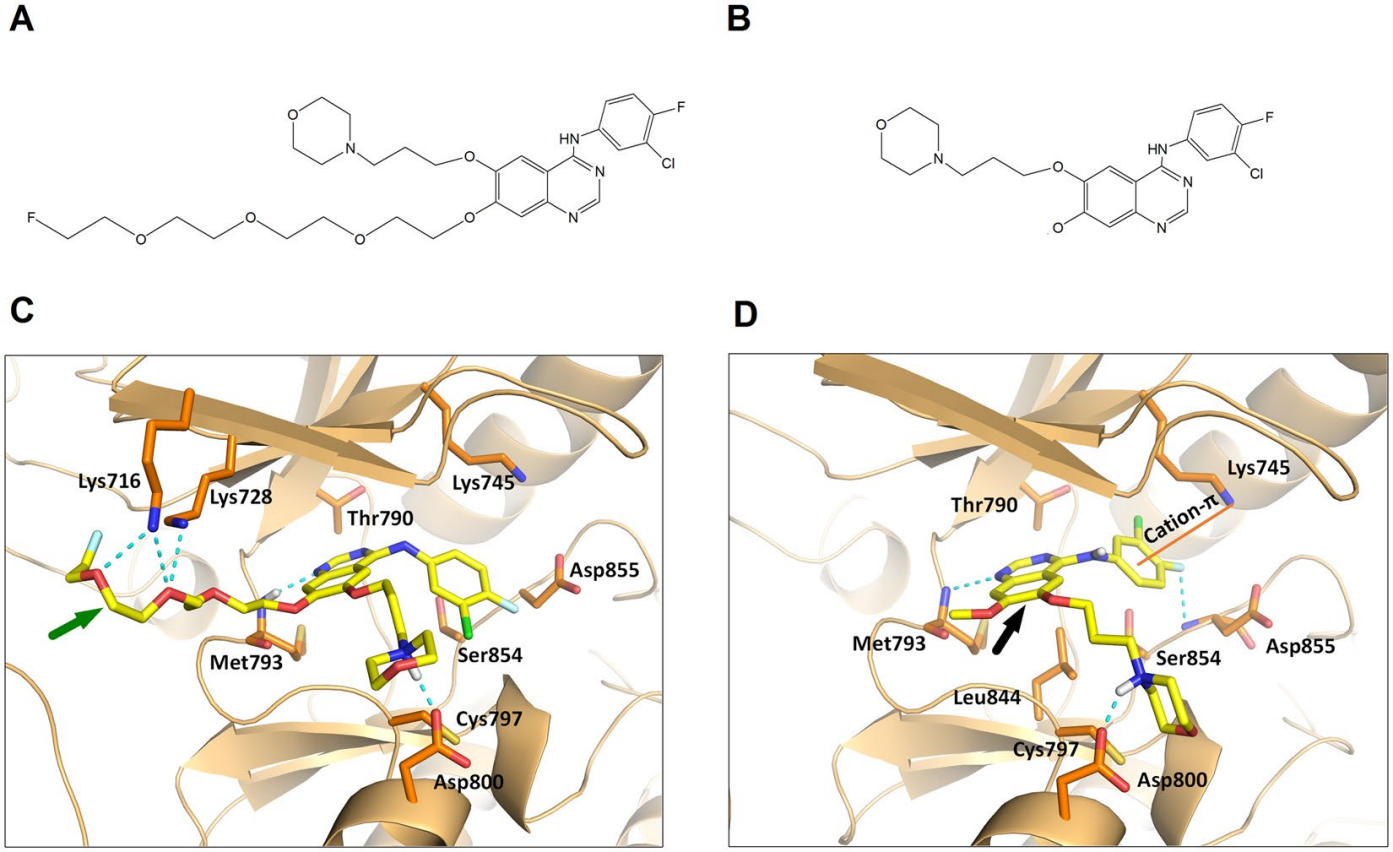
Chemical structures of F-IRS (**A**) and gefitinib (**B**). Predicted docking modes of F-IRS (**C**) and gefitinib (**D**) with EGFR 19 exon deleted structure. F-IRS (green arrow indicates F-IRS structure) binds to the receptor in a similar orientation as gefitinib (black arrow indicates gefitinib structure), maintaining interactions with Met793 and Asp800, and forming extra hydrogen bonds with Lys 745, Lys716 located in the intracellular domain of EGFR 19 exon deleted protein.

Docking results have shown that F-IRS can bind to 19 exon deleted mutant forms of the EGFR kinase domain (Fig. 1C), adopting a conformation similar to gefitinib (Fig. 1D) due to the interactions of 4-(3-chloroanilino)-quinazoline core with Met793 and Asp800. And as a result, the hydrophilic PEG chain of the molecule is externally positioned where it interacts with the outer hydrophilic surface of the binding pocket. That may contribute to the higher affinity of F-IRS than gefitinib as indicated by Glide score (F-IRS: −10.754, gefitinib: −9.482; more negative values of Glide score represent tighter binders).

### Cellular uptake, efflux and blocking experiments

**Figure 2:**
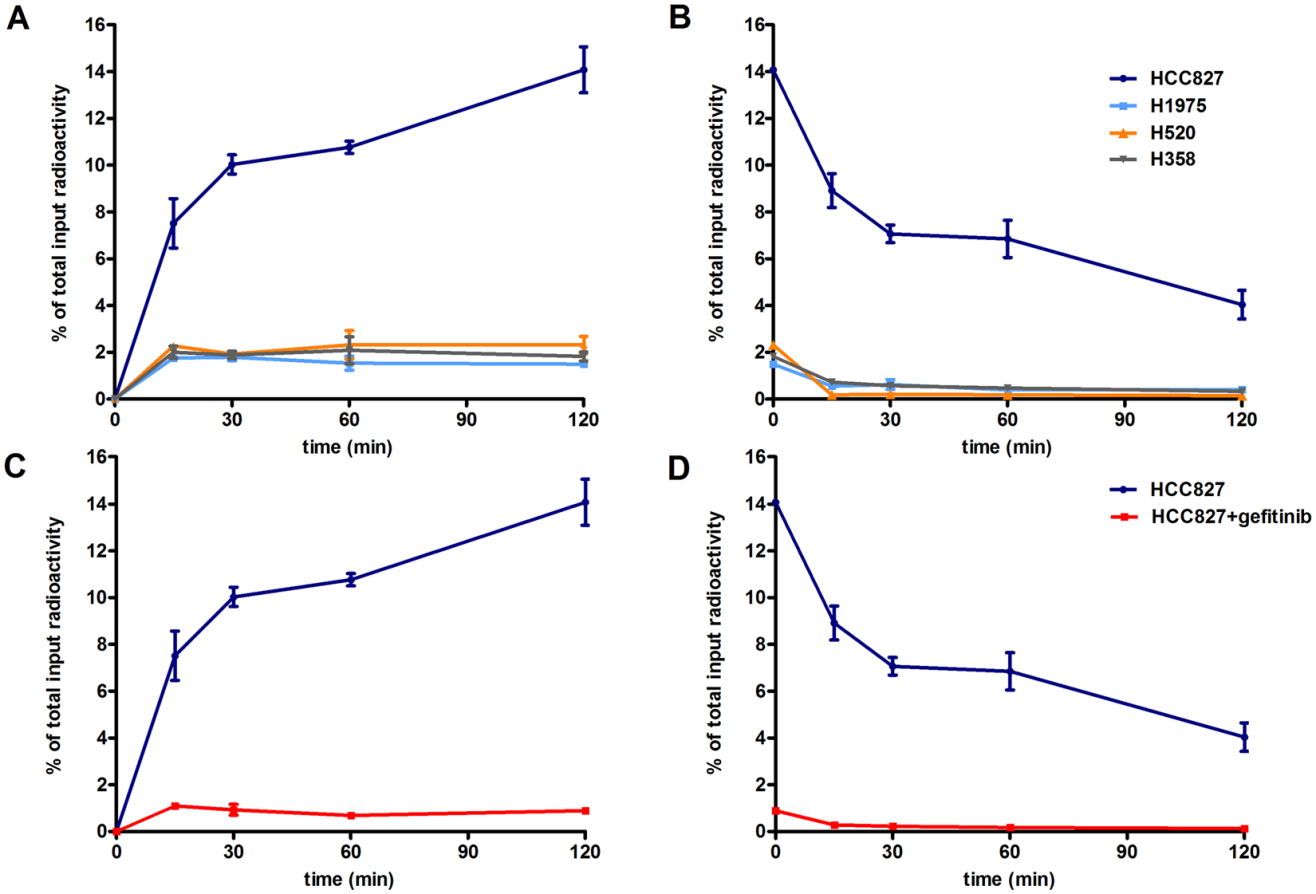
Cellular uptake and efflux of ^18^F-IRS in HCC827, H1975, H358 and H520 cell lines. (**A**) The cellular uptake of ^18^F-IRS in HCC827 was higher than others H1975, H520 and H358 cell lines at all time. (**B**) Time-dependent efflux of ^18^F-IRS in four cell lines (n = 3). Specificity of ^18^F-IRS binding with mutated EGFR in HCC827 cells. (**C**) A dramatic inhibition of cell uptake in HCC827 was observed in the presence of gefitinib (100 µmol/L). (**D**) Efflux of ^18^F-IRS in HCC827 cell line unblocked and blocked by gefitinib. Data are presented as means ± SD.

Cellular uptake assays for ^18^F-IRS were conducted to assess its specific binding to cells carrying mutations in EGFR. Rapid and significant uptake of ^18^F-IRS in HCC827 cells was observed at 15 min of incubation (7.51 ± 1.05%) reaching 10.03 ± 0.47% at 30 min of incubation (Fig. 2A). Continued exposure led to a significant increase in cellular uptake of ^18^F-IRS up to 14.07 ± 0.98% at 120 min. Accumulation of ^18^F-IRS in HCC827 cells was significantly higher than that in H1975, H358 and H520 cells (1.49 ± 0.05%, 1.82 ± 0.19%, and 2.31 ± 0.36%) after 120 min of incubation (*P* < 0.001). These results show that cellular uptake of ^18^F-IRS in HCC827 cells, but not in the other cell lines, increases with time. In addition, although eventually the retention of ^18^F-IRS in HCC827 cells decreases, it remains significantly higher than in other cell types. The efflux time point for ^18^F-IRS in HCC827 cells was at 120 min (Fig. 2B) while ^18^F-IRS efflux in the other cell lines decreased considerably at 15 min. After treating HCC827 cells with 100 μmol/L gefitinib, the cellular uptake of ^18^F-IRS dramatically decreased from 14.07 ± 0.98% to 0.89 ± 0.12% respectively (*P* < 0.001), after 120 min of incubation in both conditions (Fig. 2C). Thus, ^18^F-IRS cell uptake is strongly inhibited by gefitinib in HCC827 cells.

### Differential Sensitivity of NSCLC Cell Lines to F-IRS

*In vitro* cell growth of different NSCLC cell lines was differentially inhibited by F-IRS in a dose-dependent manner. The extremely sensitive to F-IRS were the HCC827 cells expressing 19 exon deleted mutant EGFR(IC_50_ 0.0056 ± 0.0002 μM) and the H3255 cells harboring EGFR L858R mutant (IC_50_ 0.0131 ± 0.0044 μM), whereas the H1975 cells expressing both the L858R and T790M EGFR mutations were more resistant (IC_50_ 2.27 ± 1.17 μM). The H358 expressing WT EGFR and H520 negative EGFR cells exhibited significantly resistance to (IC_50_ 7.054 ± 2.303 μM and 18.36 ± 2.21 μM) (Fig. S1).

### *In vivo* imaging and blocking experiments

**Figure 3:**
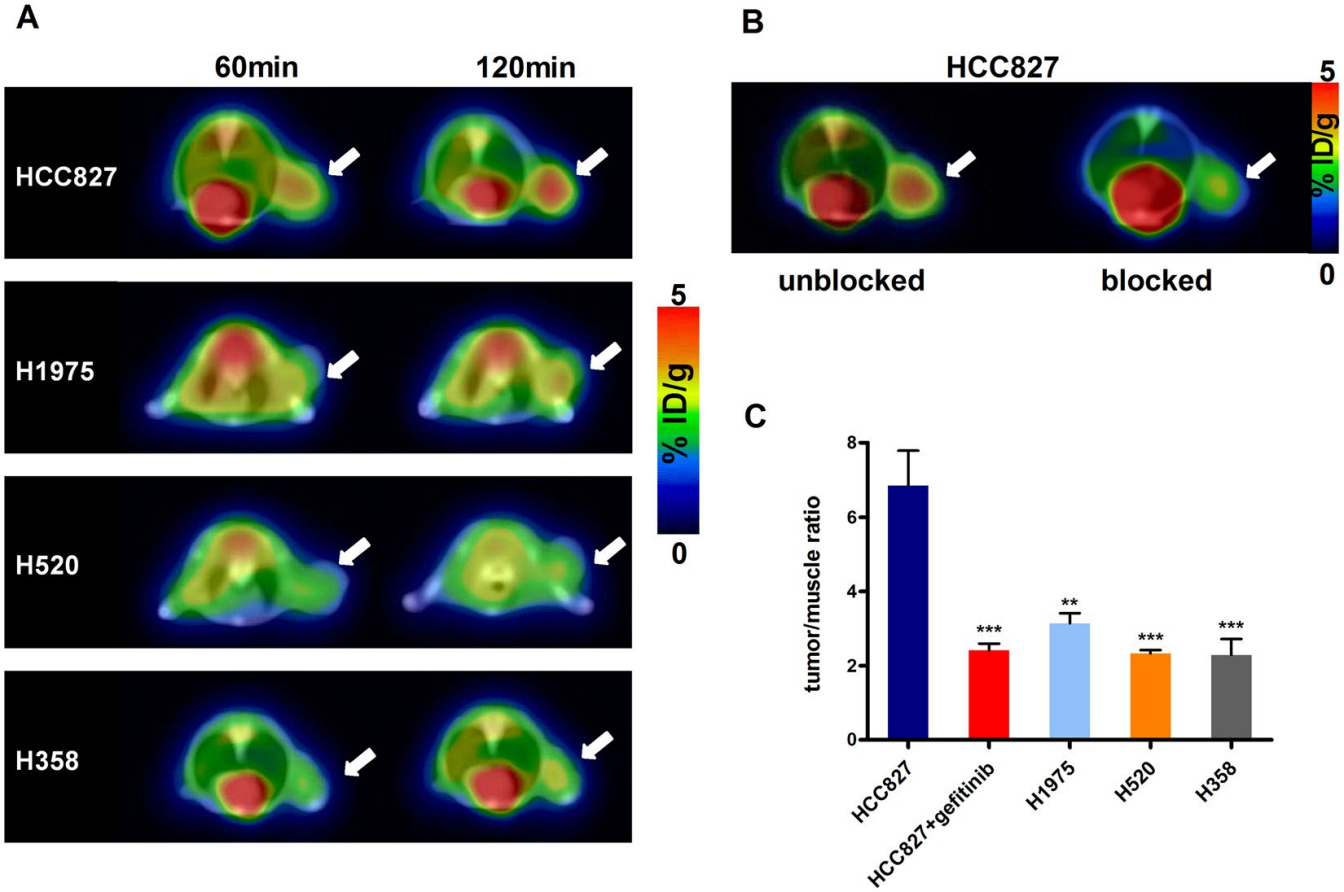
PET/CT imaging of HCC827, H1975, H358 and H520 xenografts with ^18^F-IRS. (**A**) PET/CT images of HCC827, H1975, H358 and H520 tumor–bearing mice at 60 min, and 120 min after injection of 3.7 MBq (100 μCi) of ^18^F-IRS(Axial view). Tumors (white arrow indicates tumor area) were clearly visualized after administration of ^18^F-IRS in HCC827 xenografts while the other tumor types had negligible accumulation at 60 min and 120 min time points (white arrowsindicates tumor area). (**B**) After blocked by gefitinib (100 mg/Kg), little ^18^F-IRS accumulated in the tumor (white arrow indicates tumor area) of HCC827 xenografts at 120 min. (**C**) Tumor/muscle ratios of ^18^F-IRS in HCC827, H1975, H358 and H520 xenografts at 120 min after tracer injection. Data are presented as mean ± SD. **P < 0.01 vs. HCC827 group. *** *P *< 0.001 vs. HCC827 group.

PET imaging experiments facilitated the visualization of the *in vivo* performance of ^18^F-IRS (Fig. 3A). In preliminary experiments, mice (n = 4) bearing HCC827, H1975, H358 and H520 xenografts were administered intravenously and subsequently imaged at 60 min and 120 min after injection. PET/CT images showed significantly high accumulation of ^18^F-IRS in HCC827 tumors at 120 min, however, ^18^F-IRS uptake was unobviously detectable in H1975, H520 and H358 tumors due to weak tumor uptake at all time points. In subsequent experiments, gefitinib (100 mg/kg) was injected into HCC827 xenografts nude mice (n = 4) before PET/CT imaging (Fig. 3B). The uptake ratio of ^18^F-IRS in HCC827 xenografts was blocked by gefitinib suggesting that ^18^F-IRS binds specifically to EGFR 19 exon deleted mutation. The tumor/muscle ratios of ^18^F-IRS calculated based on the PET quantification in HCC827 were significantly higher than those in H1975, H358 and H520 (*P* < 0.01) (Fig. 3C).

### Biodistribution studies

**Figure 4:**
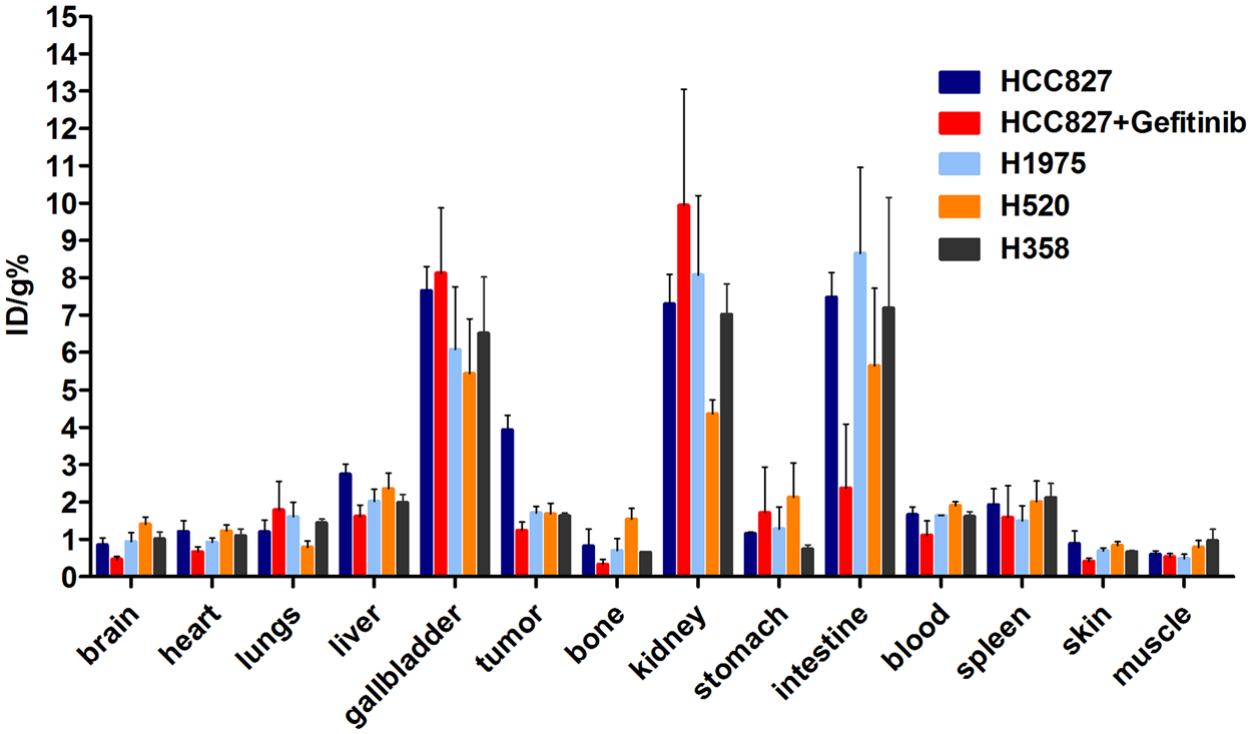
*Ex vivo* biodistribution of ^18^F-IRS (3.7 MBq per mouse) in HCC827, HCC827 blocked by gefitinib, H520, H1975 and H358 tumor-bearing nude mice at 120 min after injection. Columns mean % ID/g (n = 4 per group); error bars indicate the mean ± S.D.

In order to identify the result of PET imaging, the biodistribution studies were performed. Female nude mice bearing HCC827, H1975, H520 and H358 xenografts (n = 4 per group) were injected i.v. with 3.7 MBq (100 μCi) of ^18^F-IRS and then sacrificed at 30 min, 60 min and 120 min after injection of the tracer. The data of 120 min are expressed as the percentage injected dose per gram of tissue (% ID/g) in Fig. 4. The uptake of ^18^F-IRS in HCC827 tumors reach up to 4.27 ± 0.15% ID/g at 120 min after injection. However, in H1975, H520 and H358 tumors ^18^F-IRS uptake was significantly lower at 1.71 ± 0.178%, 1.62 ± 0.08% and 1.68 ± 0.29% at 120 min, respectively (Fig. 4). ^18^F-IRS uptake in HCC827 tumors was significantly higher than that in H1975, H520 and H358 tumors at 120 min (mean ± S.D; *P* < 0.001), which is consistent with the PET imaging. To further determine the *in vivo* binding specificity of ^18^F-IRS, we also injected gefitinib (100 mg/kg) before injection of the tracer. A significantly lower uptake of ^18^F-IRS in HCC827 xenografts blocked by gefitinib was seen compared with HCC827 xenografts unblocked (Fig. 4; 1.12 ± 0.21 ID/g vs. 4.27 ± 0.15% ID/g, *P* < 0.001) at 120 min. The uptake of ^18^F-IRS in all other organs and blood was also very low, except in the kidneys, intestine and gallbladder. There was no significant change in ^18^F-IRS uptake in other organs, including the brain, lung and skin.

### *In vitro* assays

**Figure 5:**
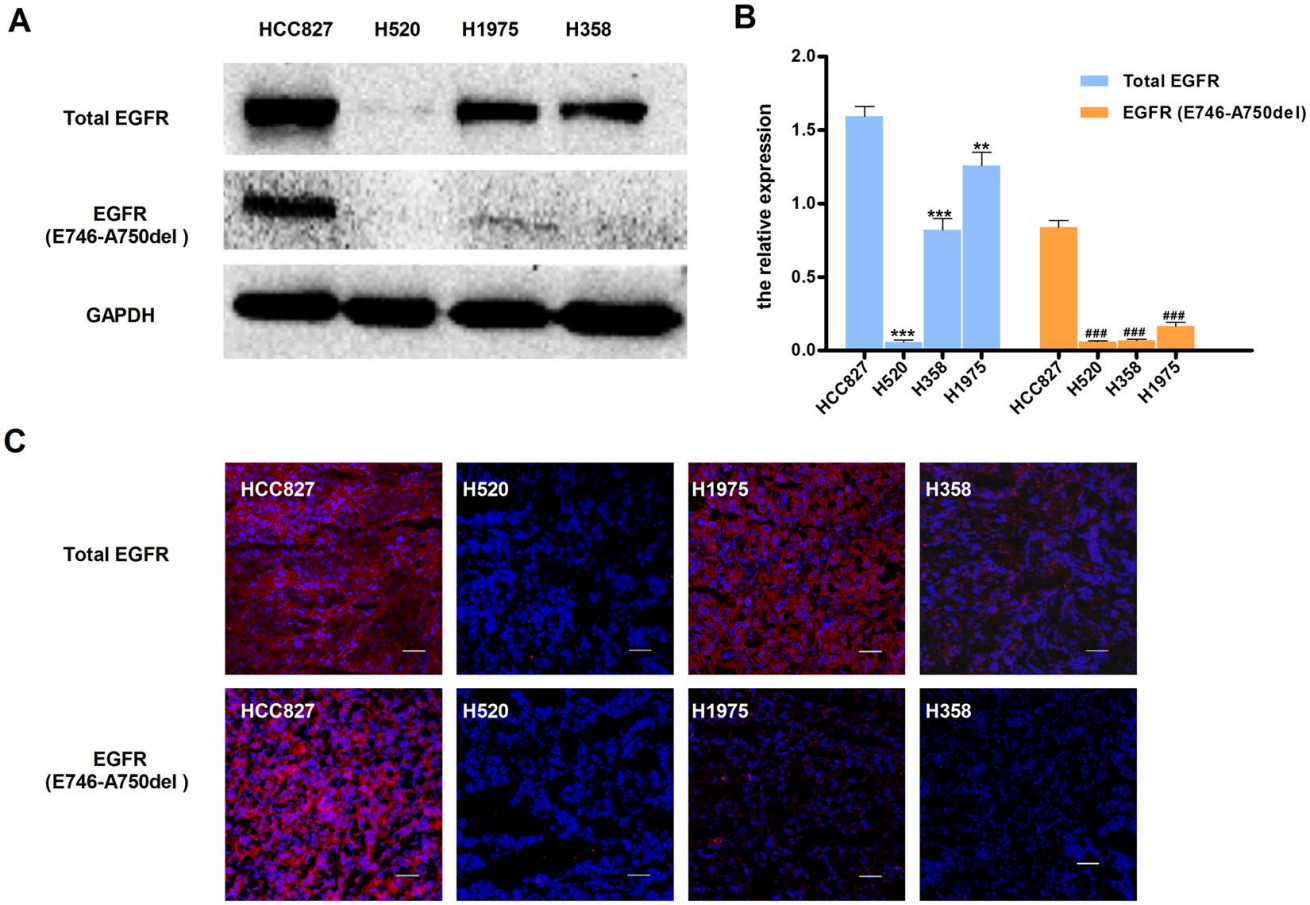
Western blot and immunofluorescence of tumors derived from HCC827, H520, H1975 and H358 cell lines. (**A**) Representative Western blots of four human NSCLC tissue lysates comparing the extent of EGFR and EGFR-specific E749-A750del mutation expression. GAPDH served as a reference for equal loading. HCC827 tissue has high EGFR and EGFR-specific E749-A750del mutation EGFR (**B**) Western blot analysis of proteins in EGFR and EGFR-specific E749-A750del mutation (n = 3). Data are presented as mean ± SD. **P < 0.01 vs. HCC827 group in total EGFR. ***P < 0.001 vs. HCC827 group in total EGFR. ^###^ *P* < 0.001 vs. HCC827 group in EGFR EGFR-specific E749-A750del mutation.(**C**) The immunofluorescence in tumors confirms western blot results. (Red color is from secondary antibody Alexa Fluor®549, and blue color from DAPI for nuclear visualization). All scale bars, 100 μm.

To confirm the expression levels and mutation status of EGFR in the four NSCLC cell lines selected for this study (HCC827, H1975, H520 and H358), western blots and immunofluorescence experiments with specific antibodies were conducted (Fig. 5). As expected, higher protein expression levels of EGFR and EGFR 19 exon E746-A750 deletion were detected in HCC827 than other three cell lines measured by western blots (Fig. 5A and B) and the results were considered statistically significant (Fig. 5B). As shown in Fig. 5C, the fluorescent signal distributed more strongly and diffusively among the whole HCC827 tumor sections than other cell lines.

### PET/CT examination of patients with NSCLC

**Figure 6:**
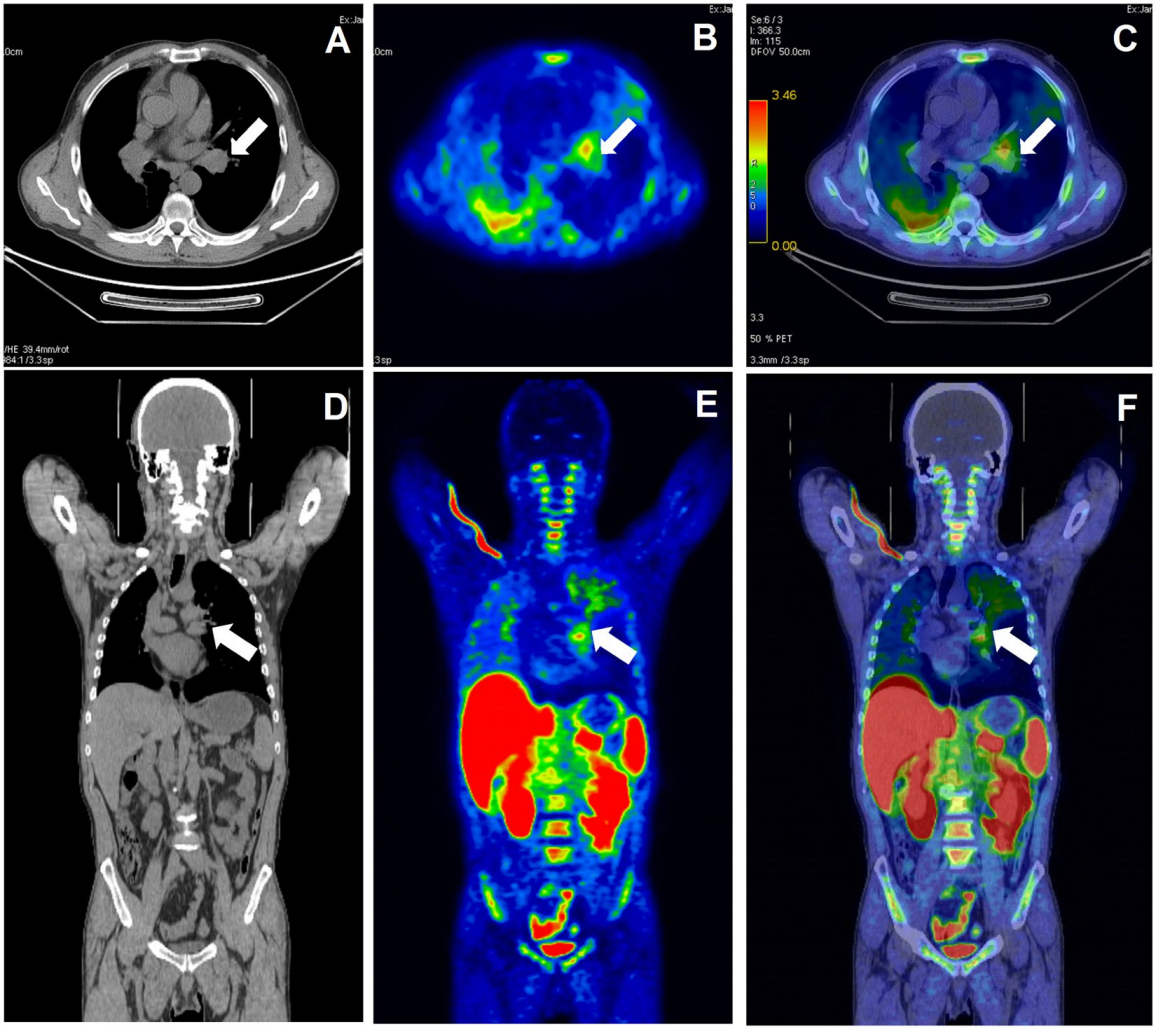
18F-IRS PET/CT scan in a patient with NSCLC of left lung. (white arrow indicates tumor area) (**A**) Transaxial CT image. (**B**) Transaxial PET image. (**C**) Transaxial PET/ CT image. (**D**) Coronal CT image. (**E**) Coronal PET image. (**F**) Coronal PET/CT image.

Injection of ^18^F-IRS caused no significant changes in heart rates, respiration rates, and blood pressure. ^18^F-IRS accumulation in tumor and organs was further quantified by measuring the Regions of Interests (ROIs) thrice and then calculating mean SUV_max_. Figure 6 shows the representative ^18^F-IRS PET/CT images of one patient with NSCLC (the images of the other two patients shown in Figs S2 and S3). Table 1 shows the average calculating mean SUV_max_ for ^18^F-IRS of the three patients participated in the study. The highest SUVs in solid organ was detected in the kidneys (SUV_max_ 13.13 ± 0.31), followed liver (SUV_max_ 10.48 ± 0.44), small intestines (SUV_max_ 8.05 ± 1.67) and bladder (SUV_max_ 5.11 ± 3.02) at 60 min after injection, indicating the renal system and the hepatobiliary system to be important in the clearance of ^18^F-IRS from the body. Low activities were found in the brain, lungs and muscles 60 min after injection, with SUV_max_ of 0.43 ± 0.29, 0.82 ± 0.23 and 0.65 ± 0.14 respectively. ^18^F-IRS accumulation was preferential in the tumor of lung (SUV_max_ 2.44 ± 0.49), which was NSCLC with EGFR exon 19 deleted.

**Table 1:** The maximum standard uptake value (SUVmax) of three patients for ^18^F-IRS.

Organ	1	2	3	Mean ± SD
Brain	0.19	0.76	0.34	0.43 ± 0.29
Lungs	0.58	1.04	0.85	0.82 ± 0.23
Tumor	2.19	2.12	3.01	2.44 ± 0.49
Kidneys	12.96	13.49	12.95	13.13 ± 0.31
Bladder	8.51	4.11	2.72	5.11 ± 3.02
Liver	10.52	10.9	10.02	10.48 ± 0.44
Small intestines	7.17	9.99	7.01	8.05 ± 1.67
Muscles	0.73	0.75	0.49	0.65 ± 0.14

### QD620-IRS flow cytometry and confocal imaging

**Figure 7:**
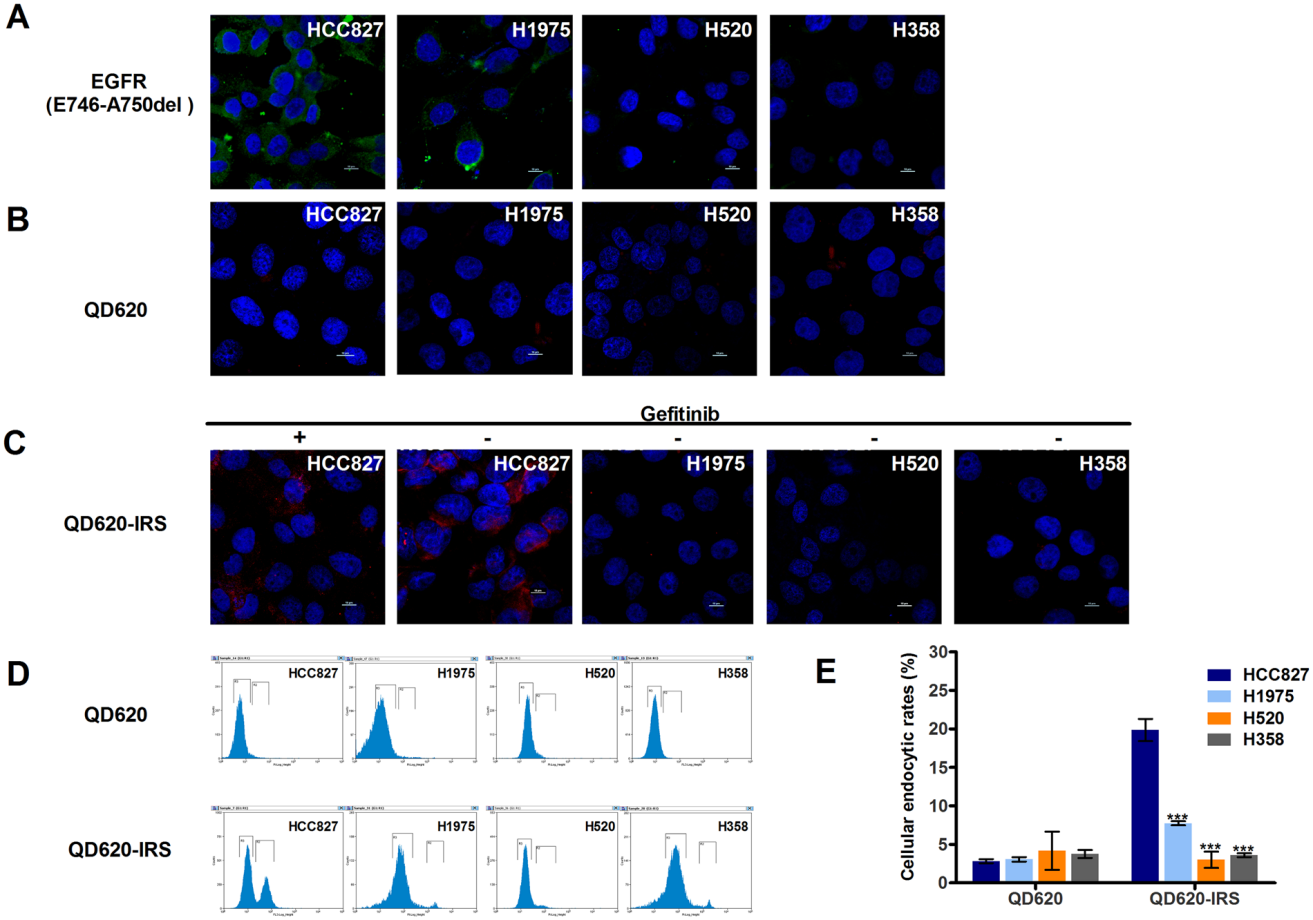
Quantitative analysis of QD620-IRS binding affinity by confocal imaging and flow cytometry. (**A**) Comparison of the expression levels of EGFR specific E749-A750del mutation in HCC827, H520, H1975 and H358 cell lines by immunofluorescence (Green color is from Alexa Fluor®488 secondary antibody, blue color from DAPI). (**B**) There is little uptake of QD620 in the four cell lines. (**C**) QD620-IRS uptake in HCC827 cells expressing EGFR 19 exon deleted mutation is considerably higher than in H1975, H520 and H358 cells. The binding of QD620-IRS in HCC827 cells was inhibited by application of gefitinib (100 μmol/L) (red circle is from QD620-IRS and blue color from DAPI). All scale bars 10 μm.(**D**) Flow cytometric analysis of endocytic rates in HCC827, H1975, H520 and H358 cells incubated with QD620-IRS or QD620 1 h. In (**E**), error bars indicate the mean ± S.D. of data from three separate experiments. ***P < 0.001 vs. HCC827 group.

We investigated the cellular uptake of QD620-IRS and QD620 on HCC827, H1975, H520 and H358 cells in a confocal microscope. The cells were darkly incubated with QD620-IRS and QD620 for 1 h. QD620 accumulation was hardly detected in HCC827, H1975, H520 or H358 cells (Fig. 7B). However, QD620-IRS uptake in HCC827 cells was visibly higher than in H1975, H520 and H358 cells (Fig. 7C), consistent with the expression of EGFR 19 exon deleted mutation (Fig. 7A). In addition, QD620-IRS uptake in HCC827 cells was inhibited by gefitinib (100 μmol/L) (Fig. 7C). To further quantify cellular uptake of QD620-IRS and QD620, the flow cytometry was performed on the same treated cells. Flow cytometry was then utilized to measure the endocytic rates of QD620-IRS and QD620. QD620 was weakly internalized in all cell lines, consistent with the confocal imaging results (Fig. 7D). In contrast, QD620-IRS was internalized very efficiently in HCC827 cells, but not in H1975, H520, and H358 cells (Fig. 7D). These results demonstrate that QD620-IRS has strong binding to EGFR 19 exon deleted mutation.

## Discussion

Non-small cell lung cancer (NSCLC) is a global public health problem of increasing importance. Since the introduction of the epidermal growth factor receptor tyrosine kinase inhibitors (EGFR-TKIs) and their approval for clinical use in the treatment of advanced NSCLC, the critical question of how to optimize EGFR-TKI therapeutic efficacy in NSCLC patients was raised^20^. A significant association between the presence of EGFR-activating mutations in lung tumors and their sensitivity to EGFR-TKIs has been well established. Thus, the diagnosis of these activating EGFR mutations that are closely associated with the therapeutic efficacy of EGFR-targeted drugs is essential for this subset of patients.

Current approaches to detect the expression and mutation status of EGFR in NSCLC include gene sequencing^21^, immunohistochemistry^22^ and FISH (Fluorescence *in situ* hybridization) analyses^23^. However, these methods can be difficult to tolerate for patients as they require repetitive biopsies of the tumor lesions. Non-invasive imaging methods are critical tools used in the management of cancer patients, in particular PET imaging, which due to its unrivalled sensitivity is highly suitable for cancer diagnosis. PET imaging of mutant EGFR is a promising non-invasive tool for real-time monitoring of EGFR expression in NSCLC patients. For instance, PET probes could not only be used for early detection of EGFR-positive tumor recurrence and stratification of cancer patients, but also for dose optimization of EGFR-TKIs therapy by monitoring the efficacy of EGFR-based tumor treatments. ^18^F is one of the most commonly used PET radioisotopes as it is widely available and has almost ideal imaging properties, making this radionuclide highly clinically relevant. Indeed, with its short half-life leading to favorable radiometric dosimetry, its high sensitivity, and its ability to quantify tissue distribution of conjugated compounds, ^18^F is an optimal PET radioisotope for clinical imaging^24^.

Here, we designed and synthesized a novel PET imaging tracer, ^18^F-N-(3-chloro-4-fluorophenyl)-7-(2(2-(2-(2-(4-fluorine)ethoxy)ethoxy)-ethoxy)-6-(3-morpholinopropoxy)quinazolin-4-amine (^18^F-IRS). While ^18^F-IRS was based on gefitinib, it showed a greater ability to bind EGFR 19 exon deleted mutation, according to molecular docking assays. Advanced synthetic strategies for the production of ^18^F-gefitinib radiolabeled 6,7-disubstituted 4- aniline-quinazolines^25^ require a multistep synthesis and are therefore time-consuming and laborious. In this study ^18^F-IRS was synthesized via a one-step radiofluorination with an efficiency of 20–30%. Importantly, the chemistry and radiochemistry of ^18^F-IRS make it suitable for clinical applications. Four polyethyleneglycol (PEG) functional groups were attached to the quinazoline ring at the 7-position to increase the water-solubility of the tracer and restructure its hydrophilicity (confirmed by the partition coefficient expressed as logP). The higher affinity of F-IRS than gefitinib to 19 exon deleted mutant forms of the EGFR kinase domain may be contributed by the hydrophilic PEG chain of ^18^F-IRS. PEG is a component that has been widely used as a drug modifier and is approved by the Food and Drug Administration of United States of America.

*In vivo* PET/CT imaging of mice and biodistribution assays showed that ^18^F-IRS is mainly eliminated through renal clearance, as evidenced by higher uptake in kidney when compared to liver. Besides having a short clearance time, ^18^F-IRS has low background level in tissues, as reflected by a low probe uptake in blood and muscle, for instance. The pharmacokinetics of ^18^F-IRS was characterized by an initial uptake in kidneys and clearance primarily through the urinary tract and to a lesser extent hepatobiliary elimination; uptake in heart, lungs and blood was low with rapid clearance of the probe. Although increasing the water-solubility of the probe, part of ^18^F-IRS was uptaken into hepatobiliary, especially intestine and gall-bladder. We will optimize the probe to increase the water solubility in order to improve radiotracer pharmacokinetic characteristics in the following study. Our pilot study of three patients with NSCLC showed that there was a significant difference in ^18^F-IRS uptake between the tumor mass and healthy tissue. The data of patients with NSCLC demonstrated that the main route of elimination of the tracer was the urinary tract, followed by the hepatobiliary, a pattern of excretion similar to that in the mouse model. Limited by the number of patients, only three patients with NSCLC harboring EGFR 19 exon deleted mutation were recruited in this study. In order to assess ^18^F-IRS specific binding to patients carrying mutations in EGFR on clinical, we will recruit patients with NSCLC harboring various EGFR mutation, such as L858R mutant EGFR, WT EGFR and L858R/T790M dualmutant EGFR.

The specific binding ability of ^18^F-IRS to EGFR-activating mutation (EGFR 19 exon deleted mutation) was evaluated by *in vitro* cell assays, flow cytometry and confocal imaging, as well as *in vivo* PET/CT imaging studies and *ex vivo* biodistribution studies. Cellular uptake and efflux experiments showed that ^18^F-IRS uptake was significantly higher in HCC827 cells than H1975, H520 or H358 cells, revealing that the probe binds specifically to the EGFR 19 exon deleted mutation. Furthermore, ^18^F-IRS uptake in HCC827 cells was significantly reduced by the addition of gefitinib to the culture medium, further showing the high specificity of the probe to the EGFR-activating mutation. Consistent with these results, ^18^F-IRS showed a higher accumulation in HCC827 xenografts (when compared to the other cell lines) that was also blocked by gefitinib. Although H1975 cells also carry the L858R EGFR mutation, the second T790M mutation causes resistance to EGFR inhibitors such as gefitinib and erlotinib by interfering with their binding to EGFR^26, 27^. Specifically, the T790M mutation is predicted to sterically block inhibitor binding via the methionine side chain as the quinazoline ring for EGFR inhibitors^28^. Western blot and immunofluorescence analyses confirmed that HCC827 tumors expressed high levels of EGFR and EGFR 19 exon deleted mutation but H1975, H358, and H520 had nearly no expression of the activating EGFR mutation, further suggesting that ^18^F-IRS has a higher binding affinity to the EGFR mutation sensitive to gefitinib. Although the number of patients in this study was limited, together these results demonstrate that ^18^F-IRS is a valuable PET probe for the specific detection of tumors bearing EGFR-activating mutations and as a guide for personalized therapy in NSCLC.

## Conclusion

We have successfully developed a radiotracer labeled with ^18^F that targets tumors carrying an EGFR 19 exon deleted mutation. Notably, ^18^F-IRS shows high stability and specificity to EGFR 19 exon deleted mutation not only *in vitro* but also *in vivo* in NSCLC xenografts. Thus, PET/CT imaging with ^18^F-IRS could potentially become a new approach for diagnosing NSCLC molecular-genetic patient subtypes as well as other EGFR-driven tumor types. Finally, this non-invasive method could be used for selecting patients for individualized therapy with EGFR inhibitors and for monitoring the efficacy of these treatments.

## Methods

### Preparation of ^18^F-IRS

Synthesis and quality control of ^18^F-IRS was performed as previously described^29^. Radio-synthesis of ^18^F-IRS was performed on a GE TRACERlabFN (GE Healthcare, USA) equipped with an integrated HPLC system.

### Preparation and application of QD620-IRS

Quantum dots QSA-620 (a gift from Yongqiang Wang in Ocean NanoTech company) is a water-soluble alloy CdSSe/ZnS quantum dots with amphiphilic polymer and PEG coating. The reactive group is amine. QD620-IRS complexes were formed by 400 μl of sodium tetraborate (50 mM, pH 8.5) mixed with 200 μl IRST (6.5 mM, approximately 1 mg) and 125 μl QSA-620 (8 μM). Before adding an excess of DMSO, the complexes of QSA-620 and IRST were stirred and heated at 80 °C for 15 min in a reaction vessel. After centrifugation at 13500 RPM for 5 min, the supernatant was removed and the precipitate was re-dissolved in 1 ml of water, then stored at 4 °C before confocal imaging was performed.

### Molecular Docking

Glide embedded in SchrÖdinger 10.1 Suite^30^ was employed to carry out molecular docking. Structures of cytoplasmic kinase domain of EGFR were downloaded from Protein Data Bank. Homology modeling is performed with Discovery Studio 3.5^31^ to build 19del-EGFR structure. Gefitinib-bound EGFR structure (2ITY) was selected as the template. To prepare the protein structure before docking, solvent molecules were deleted and missing hydrogens atoms were added. Considering the large size of F-IRS, the ligand-binding region was defined by a 30 Å box centered on the ligand from the crystal structure. The structures of F-IRS and Gefitinib were sketched using Maestro and was energy-minimized under MMFFs force field. Rigid receptor-flexible ligand docking was performed using Glide XP module.

### Cell Culture

Four human NSCLC cell lines with different EGFR expression levels and mutational status were selected to determine whether ^18^F-IRS can specifically accumulate in EGFR-mutation tumors: HCC827 (EGFR 19 exon deleted mutation), H1975 (EGFR L858R/T790M mutation), H358 (EGFR wild type) and H520 (negative EGFR). HCC827 cells carry a deletion in the exon 19 of EGFR that makes them highly sensitive to EGFR-TKIs treatment^4^. In contrast, H1975 harbors both the activating L858R mutation and the T790M secondary mutation, which interferes with the binding of gefitinib to the EGFR kinase domain thus conferring resistance to gefitinib^32^. The H358 cell line is less sensitive to gefitinib^33^ and H520 cells do not respond to gefitinib^34^. All cell lines were obtained from the ATCC (American Type Culture Collection). The cells were cultured in RPMI 1640 medium (HyClone) supplemented with 10% fetal bovine serum (HyClone) and 1% penicillin–streptomycin (HyClone) and maintained at 37 °C, 5% CO_2_ in a humidified incubator.

### Cellular uptake, efflux and blocking assays

Cells were trypsinized and seeded overnight in 12-well culture plates at a density of 1 × 10^5^ cells per well (1 × 10^5^ cells/1.0 mL/well). ^18^F-IRS (37 kBq/1.0 mL/well) in 1 ml of serum free 1640 medium was added to each well and incubated at 37 °C for 15 min, 30 min, 60 min and 120 min. Triplicates were carried out for each time point. One milliliter of ice-cold PBS was used to intercept the uptake of tracer. The supernatants were aspirated and the cells were rinsed twice with 1 mL of ice-cold PBS to remove surface-bound radioactive ligand. After washing twice with PBS, the cells were harvested by the addition of 250 μl of 0.1 N NaOH. Efflux assays were performed after 120 min of incubation with ^18^F-IRS and then the media was removed and replaced with serum free 1640 medium without ^18^F-IRS. At 15 min, 30 min, 60 min and 120 min, the media was removed and the cells washed twice with ice-cold PBS. 200 μl of 0.1 N NaOH were added to dialyse the cells and then the wells were washed twice with 1 ml of ice-cold PBS. The cell solution and the PBS from each washed well were recovered and counted with CPM in a gamma counter (Wizard 2, PerkinElmer). The results were calculated using the same cellular uptake formula. For blocking experiments, gefitinib (100 μmol/L) was added to HCC827 cells. After 60 min of incubation, the uptake and efflux assays in HCC827 cells were repeated thrice.

### MTT assay

NSCLC having EGFR with L858R mutation is also respond to EGFR kinase inhibitors. H3255 cells expressing EGFR L858R mutant is sensitive to EGFR kinase inhibitors^17^. In order to measure the sensitivity of five NSCLC cell lines to F-IRS, MTT(dimethyl sulfoxide) assay was performed. Tumor cells were plated at a density of 2 × 10^3^ cells/well into 96-well plates in RPMI 1640 with 10% FBS. Cells were kept in the log phase of proliferative activity. Various concentrations of F-IRS added to each well, and incubation was continued for an additional 48 h. Then, 50 μL of MTT solution (2 mg/mL; Sigma) were added to all wells, and incubation was continued for another 2 h. The media containing MTT solution was removed, and the precipitated material was dissolved by adding 100 μL of DMSO. The absorbance was measured with a microplate reader at test and reference wavelengths of 490 nm, respectively. The percentage of growth is shown relative to untreated controls.

### Animal xenograft models

All animal studies were conducted in accordance with the China Guidelines for Animal Care and Ethic for Animal Experiments and the experimental protocols were approved by the Animal Use and Care Committee of Harbin Medical University. Female BALB/c nude mice (from the Shanghai Slack Experimental Animal Center of qualified animals SCXK) approximately 4–6 weeks of age and mean weight 20–22 g were used. Approximately 5 × 10^6^ of cultured HCC827, H1975, H358 and H520 cells were suspended in PBS/Matrigel (1:1) (BD Bioscience) and subcutaneously implanted in the right shoulders of Female BALB/c nude mice. PET/CT studies were performed when the tumor volume reached 250 mm^3^ (4–6 weeks after inoculation).

### *In vivo* PET/CT imaging and blocking experiments of mice

PET/CT imaging of tumor-bearing mice was performed on a clinical time-of-flight (TOF) 64-slice PET/Computed tomography (CT) scanner (Discovery 790 Elite, GE healthcare). The mice bearing tumors (for each group n = 4) were injected with 3.7 MBq (100 μCi) of ^18^F-IRS into the tail vein. At 30 min, 60 min and 120 min after injection, the mice were anesthetized with 2% isoflurane and placed near the center of the field of view of the PET/CT scanner in prone position. For blocking studies, gefitinib (100 mg/kg) was injected into HCC827 xenografts nude mice (n = 4) via tail vein 60 min before PET/CT imaging. The mice were imaged as described above 120 min after injection.

The images were reconstructed using a two-dimensional ordered-subset expectation maximization (OSEM) algorithm. For each scan, regions of interest (ROIs) were drawn over the tumor and major organs using the AW4.6 software (GE Healthcare) on decay-corrected whole-body coronal images. The radioactivity concentrations (accumulations) within the tumor, muscle were obtained from mean pixel values within the multiple ROI volumes and then converted to megabecquerels per milliliter per minute using the calibration factor determined for the PET system. These values were then divided by the administered activity to obtain (assuming a tissue density of 1 g/ml) an image ROI-derived percent injected dose per gram (% ID/g).

### Biodistribution of ^18^F-IRS in Tumor Models

Biodistribution studies were performed in BALB/c nude mice bearing HCC827, H1975, H358 and H520 xenografts by injecting 3.7MBq of ^18^F-IRS into the tail vein (n = 4 per group) and then sacrificing the animals at 30 min, 60 min and 120 min after injection. Tumor and normal tissues were excised and weighed, and the radioactivity was measured using a gamma-counter (Wizard 2, PerkinElmer). All measurements were background-subtracted and decay-corrected to the time of injection and then averaged together. The radioactivity uptake in tumor and normal tissues was expressed as the percentage of the injected dose per gram of organ (% ID/g).

### Western Blot

After radioactivity decay following the PET/CT studies, the mice were sacrificed and the tumor tissues harvested for western blots. Tumor tissue proteins were extracted with extraction buffer and their concentration was determined using a BCA protein assay kit (Pierce Biotechnology, Inc.). After sodium dodecyl sulfate polyacrylamide gel electrophoresis separation of 90 ug of total protein, the proteins were transferred to a nitrocellulose filter membrane (NC) membrane and incubated at room temperature with 5% nonfat milk blocking buffer. The blots were then incubated overnight at 4 °C with EGF Receptor (D38B1) Rabbit mAb antibody (1:1000 Cell Signaling) and EGF Receptor (E746-A750 Specific) Rabbit mAb antibody (1:1000; Cell Signaling), followed by incubation at room temperature for 2 h with anti-rabbit IgG, HRP-linked antibody (Cell Signaling) or anti-mouse IgG, HRP-linked antibody (Cell Signaling) in 5% BSA/TBST. The protein bands were detected using the ECL Western blotting detection system (BD). GAPDH was used as a loading control. After development, the films were scanned with BIO-RAD Gel Doc XR+. The images were opened and analyzed by ImageLab(BIO-RAD) software. Three samples of each tumor model were prepared for western blot to obtain semiquantitative data for statistical analysis.

### Immunofluorescence

Frozen tumor tissue slices (5 μm) were fixed with cold 4% paraformaldehyde (PFA) for 30 min, and then washed with PBS three times. After blocking with TBS 0.1% Tween-20 (TBST)/10% normal goat serum for 1 h, the sections were incubated with EGF Receptor (D38B1) Rabbit mAb antibody (1:50; Cell Signaling) and EGF Receptor (E746-A750 Specific) Rabbit mAb antibody (1:50; Cell Signaling) at 4 °C overnight and then covered with fluorescein isothiocyanate-conjugated (FITC) secondary antibody Goat anti-Rabbit IgG (H + L) Secondary Antibody, Alexa Fluor® 594 conjugate (1:1000; ThermoFisher). After being washed three times with PBS, the slices were mounted with 49-6-diamidino-2-phenylindole (DAPI)-containing mounting medium and observed under a confocal microscope. Each type of tumor tissue slices was prepared in triplicates.

### PET/CT examination of patients with NSCLC

This study (ClinicalTrials.gov number, NCT03031522) was approved by the Medical Ethics Review Committee of the Fourth Hospital of Harbin Medical University. Subjects signed written informed consent after receiving an explanation of the study. The images provided in this article have been agreed by the subjects. The diagnosis was confirmed by histopathological examination. Three patients confirmed having NSCLC with responsive EGFR exon 19 deleted by histopathology participated in the study from January 2015 to February 2016 (Table 2). ^18^F-IRS scans were performed on PET/CT (Discovery 790 Elite; GE healthcare). Approximately 222MBq (6 mCi) of ^18^F-IRS was administered intravenously within 5 minutes. Scanning was in initiated 1 hour after administration of ^18^F-IRS. Corresponding whole body CT scans for attenuation correction were acquired using a low-dose protocol. Consecutively, PET emission data were acquired in three-dimensional mode with a 200 × 200 matrix with 3 min emission time per bed position. The maximum standard uptake value (SUV_max_) normalized to body weight (kBq/mL) was calculated within the region of interest with the following formula: [measured activity concentration (Bq/mL) · body weight (kg)]/injected activity (Bq). Calculation of the radioactivity concentration included the use of a calibration factor that converted the activity counts into becquerels, and the decay correction of the Advantage Workstation 4.6 workstation (GE Healthcare) was removed.

**Table 2:** Subject Characteristics.

Subject or parameter	Age (y)	Height (cm)	Weight (kg)	Injected dose (MBq)	Sex
1	49	165	65	240.5	M
2	70	178	87.5	323.7	M
3	57	172	59.9	222	F

### Determination of QD620-IRS binding specificity by confocal imaging and flow cytometry

QD620-IRS binding specificity was quantified by confocal imaging and flow cytometry. For confocal imaging, HCC827, H1975, H520 and H358 cells were grown in Lab-Tek chamber slides and then washed twice with PBS, fixed with 4% paraformaldehyde for 15 min, permeabilized with 0.5% Triton X-100 for 15 min followed by blocking with 10% bovine serum albumin for at least 1 h. The cells were stained with either QD620-IRS or QD620 (750 nM) for 60 min at room temperature in the dark, or with EGFR (E746-A750 Specific) Rabbit mAb antibody (1:250; Cell Signaling) at 4 °C overnight. They were then stained with with FITC secondary antibody Goat anti-Rabbit IgG (H + L) Secondary Antibody or Alexa Fluor®488 conjugate (1:1000; ThermoFisher). After being washed with PBS, all cells were mounted with DAPI-containing mounting medium and observed with a confocal microscope (C2Si, Nikon). To confirm that QD620-IRS binds to 19 exon deletion mutant EGFR, fixed HCC827 cells were blocked with 10% BSA for 30 min and then incubated with gefitinib (100 μmol/L) for 1 h at room temperature, followed by incubation with QD620-IRS (750 nmol/L) for 1 h at room temperature. After washing the cells were fixed and mounted with DAPI-containing mounting medium for observation with a confocal microscope (C2Si, Nikon). For flow cytometry, trypsinized HCC827, H1975, H520 and H358 cells were fixed with cold 4% paraformaldehyde (PFA) for 30 min and then washed with PBS three times. After permeabilization with 0.1% Triton-100 for 5 min, the cells were labeled with QD620-IRS or QD620 (750 nM) for 60 min at room temperature, washed multiple times and then analyzed on a flow cytometer (MoFloTM XDP, Becton Dickinson).

### Statistical analysis

Data are expressed as Means ± SD. Statistical analyses were carried out using a statistics program (GraphPad Prism 5.0). For multiple comparisons, a one-way analysis of variance (ANOVA) followed by the Bonferroni’s multiple comparisons test was used. *P*-value of <0.05 was considered statistically significant.

## Electronic supplementary material

Supplementary information
