# Author Correction: Development and Evaluation of ^18^F-IRS for Molecular Imaging Mutant EGF Receptors in NSCLC

**DOI:** 10.1038/s41598-021-99707-w

**Published:** 2021-10-14

**Authors:** Yan Song, Zunyu Xiao, Kai Wang, Xiance Wang, Chongqing Zhang, Fang Fang, Xilin Sun, Baozhong Shen

**Affiliations:** 1 Molecular Imaging Research Center, Harbin Medical University, Harbin, Heilongjiang China; 2 TOF-PET/CT/MR Center, The Fourth Hospital of Harbin Medical University, Harbin, Heilongjiang China; 3 Molecular Imaging Program at Stanford (MIPS), Department of Radiology, Stanford University School of Medicine, Stanford, California USA

Correction to: *Scientific Reports* 10.1038/s41598-017-01443-7, published online 09 June 2017

This Article contains errors.

In Figure 5A, the blots for EGFR (E746-A750del) and GAPDH band are incorrect, which affected the quantification in Figure 5B. A corrected version of Figure 5 and its accompanying legend appear below.

**Figure 5:**
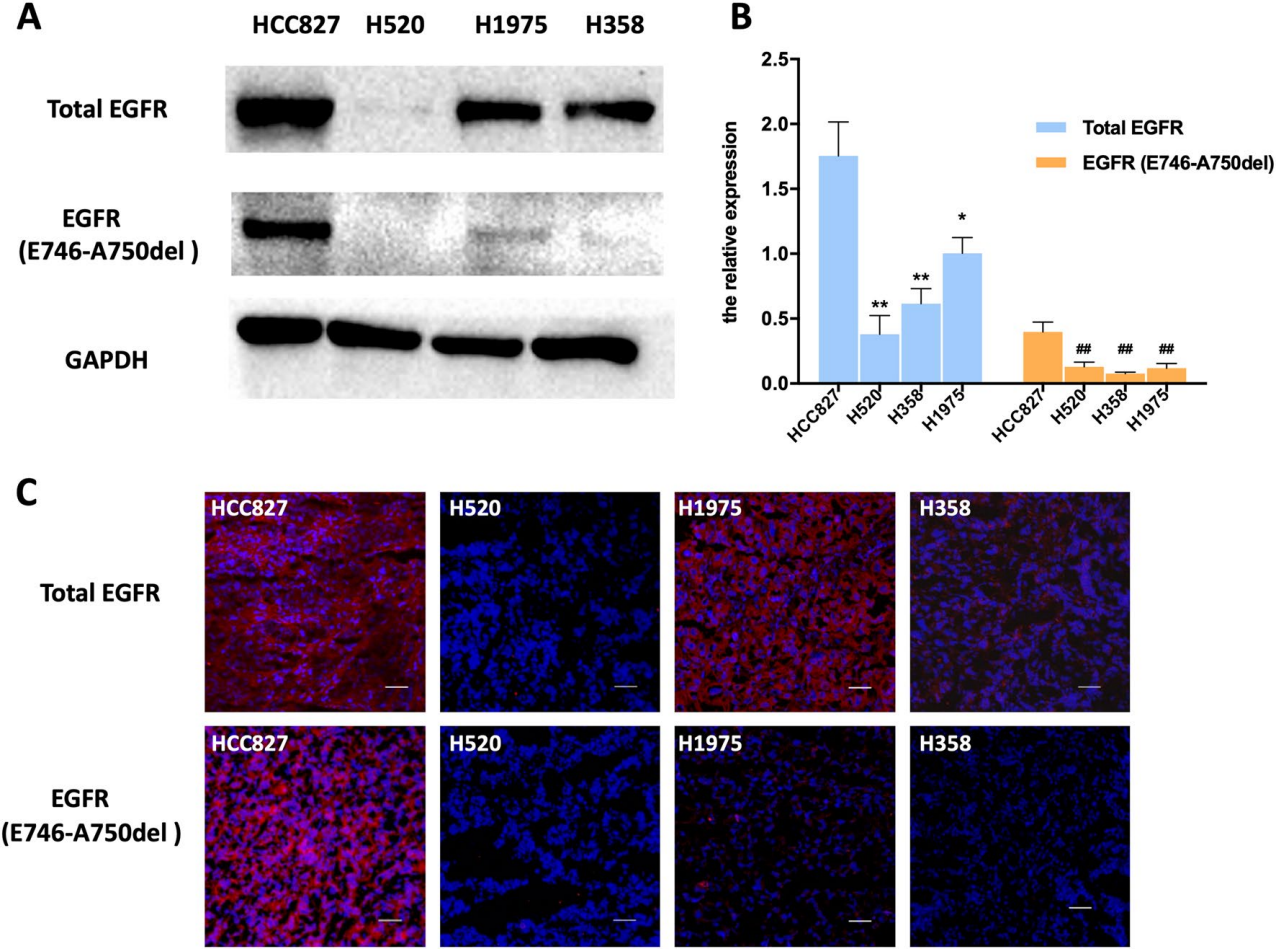
Western blot and immunofluorescence of tumors derived from HCC827, H520, H1975 and H358 cell lines. (**A**) Representative Western blots of four human NSCLC tissue lysates comparing the extent of EGFR and EGFR-specific E749-A750del mutation expression. GAPDH served as a reference for equal loading. HCC827 tissue has high EGFR and EGFR-specific E749-A750del mutation EGFR (**B**) Western blot analysis of proteins in total EGFR and EGFR-specific E749-A750del mutation (n = 3). Data are presented as mean ± SD. *P < 0.05 vs. HCC827 group in total EGFR. **P < 0.01 vs. HCC827 group in total EGFR. ^##^*P* < 0.01 vs. HCC827 group in EGFR-specific E749-A750del mutation. (**C**) The immunofluorescence in tumors confirms western blot results. (Red color is from secondary antibody Alexa Fluor®549, and blue color from DAPI for nuclear visualization). All scale bars, 100 μm.

In Figure 7B, the image for the H358 is incorrect. The correct Figure 7 appears below with its accompanying legend.

**Figure 7:**
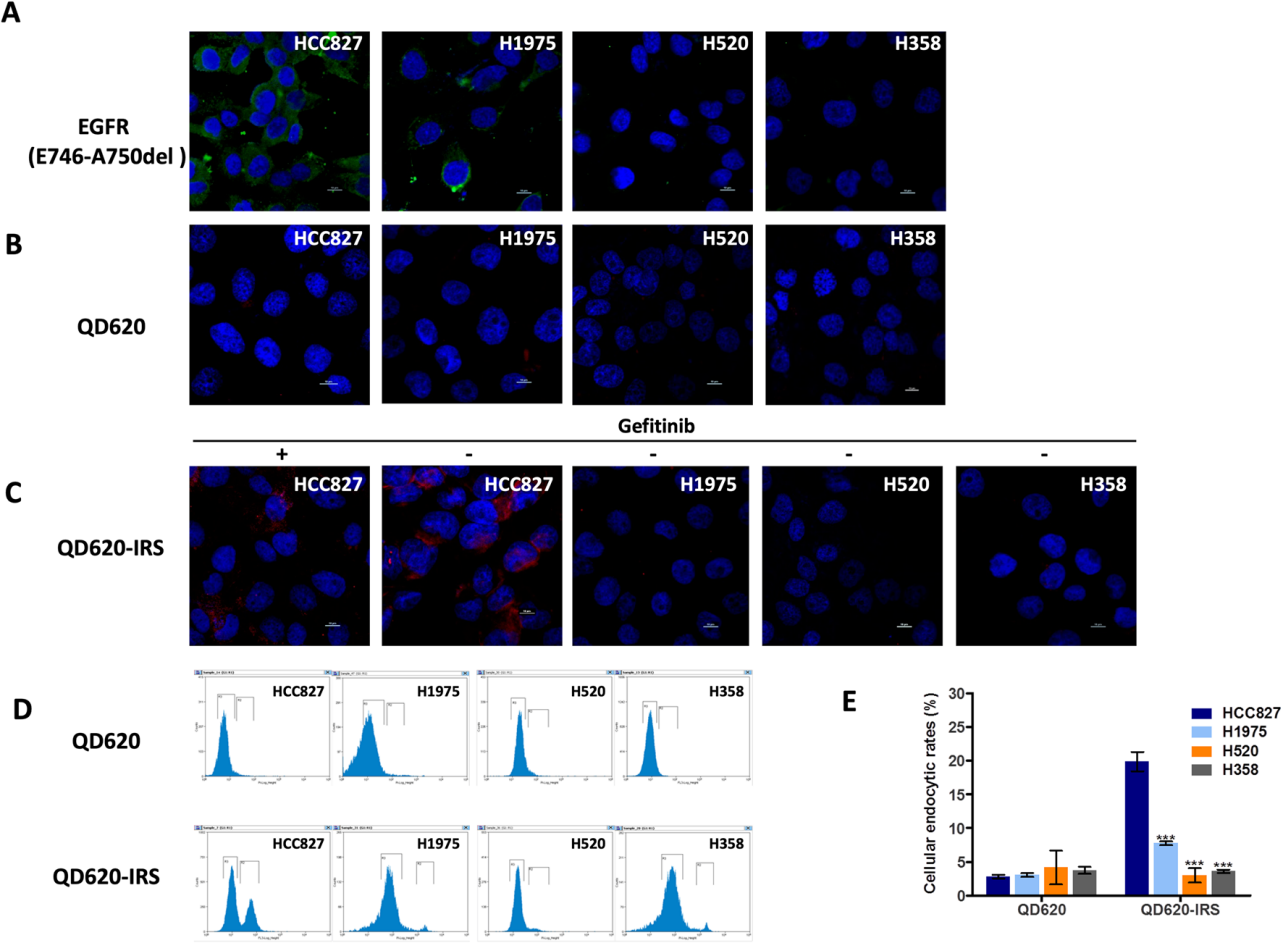
Quantitative analysis of QD620-IRS binding affinity by confocal imaging and flow cytometry. (**A**) Comparison of the expression levels of EGFR specific E749-A750del mutation in HCC827, H520, H1975 and H358 cell lines by immunofluorescence (Green color is from Alexa Fluor®488 secondary antibody, blue color from DAPI). (**B**) There is little uptake of QD620 in the four cell lines. (**C**) QD620-IRS uptake in HCC827 cells expressing EGFR 19 exon deleted mutation is considerably higher than in H1975, H520 and H358 cells. The binding of QD620-IRS in HCC827 cells was inhibited by application of gefitinib (100 μmol/L) (red circle is from QD620-IRS and blue color from DAPI). All scale bars 10 μm. (**D**) Flow cytometric analysis of endocytic rates in HCC827, H1975, H520 and H358 cells incubated with QD620-IRS or QD620 1 h. In (**E**), error bars indicate the mean ± S.D. of data from three separate experiments. ***P < 0.001 vs. HCC827 group.

This correction does not alter the conclusions of this study.

