# Supplementary material for: Development and Evaluation of 18F-IRS for Molecular Imaging Mutant EGF Receptors in NSCLC
Source: Sci Rep. 2017 Jun 9;7:3121. doi: 10.1038/s41598-017-01443-7 (PMC5466683; doi:10.1038/s41598-017-01443-7)
Supplement: Supplementary file 1 — Supplementary information [file 41598_2017_1443_MOESM1_ESM.doc]

**Development and Evaluation of ^18^F-IRS for Molecular Imaging Mutant EGF Receptors in NSCLC**

**Yan Song^#1,2^, Zunyu Xiao^#1,2^, Kai Wang^1,2^, Xiance Wang^1,2^, Chongqing Zhang^1,2^,Fang Fang^1,2^,**

**Xilin Sun*^1,2,3^, Baozhong Shen*^1,2^**

^1^Molecular Imaging Research Center, Harbin Medical University, Harbin, Heilongjiang, China

^2^TOF-PET/CT/MR center, The Fourth Hospital of Harbin Medical University, Harbin, Heilongjiang, China

^3^Molecular Imaging Program at Stanford (MIPS), Department of Radiology, Stanford University School of Medicine, Stanford, California, USA.

#These authors contributed equally to this work and they should be considered as the first author. Correspondence and requests for materials should be addressed to Baozhong Shen (email: shenbzh@vip.sina.com) or Xilin Sun (email: sunxilin@aliyun.com)

**Fig. S1**

**
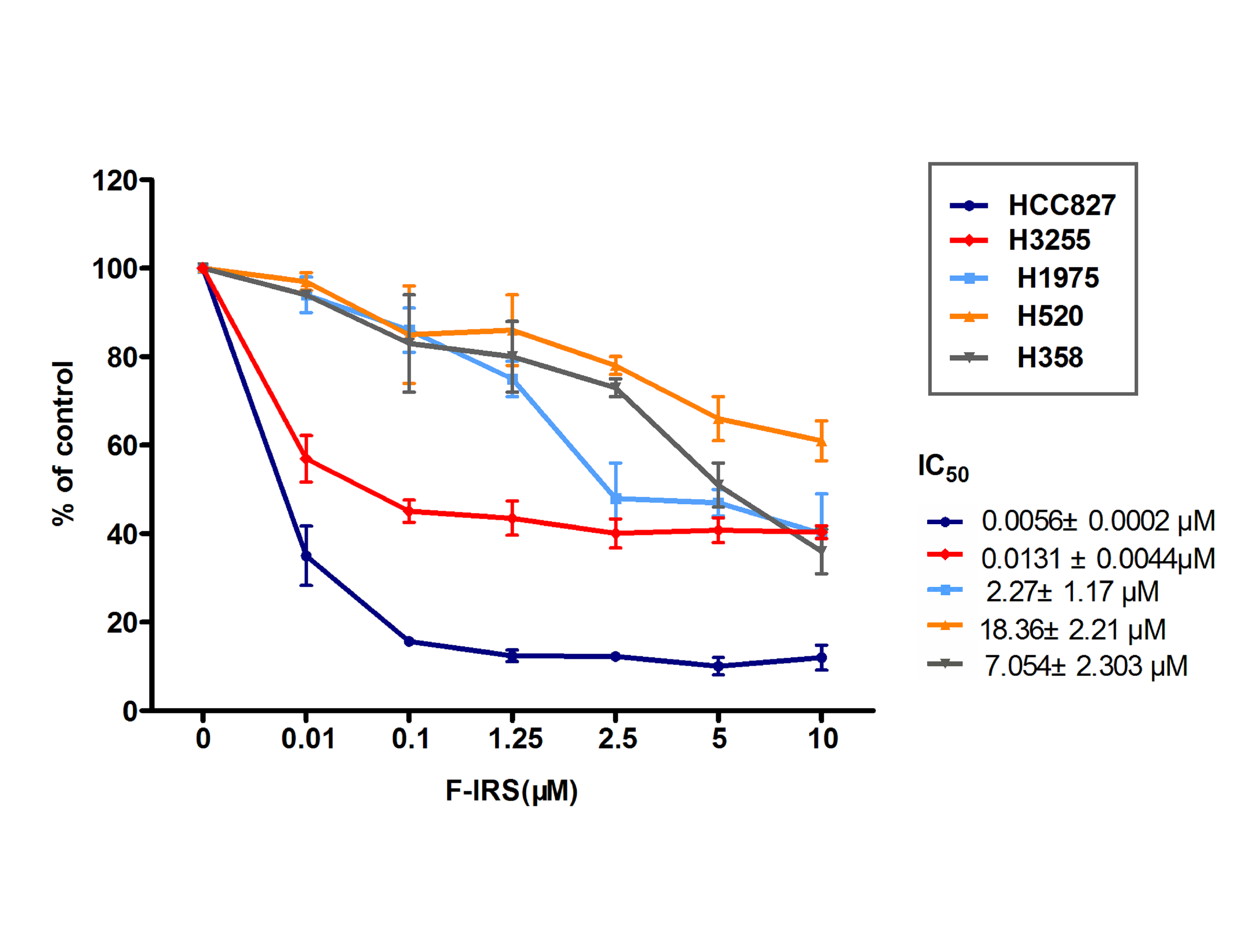
**

Cell growth inhibition by F-IRS treatment. Cells were treated with the indicated concentration of F-IRS for 48h in complete medium. MTT assay was used to assess growth of cells in culture. Percent of cell growth (percent of control) was expressed as mean ± SD value of percentage of absorbance reading from treated cells vs. untreated cells from triplicate wells.

**Fig. S2**


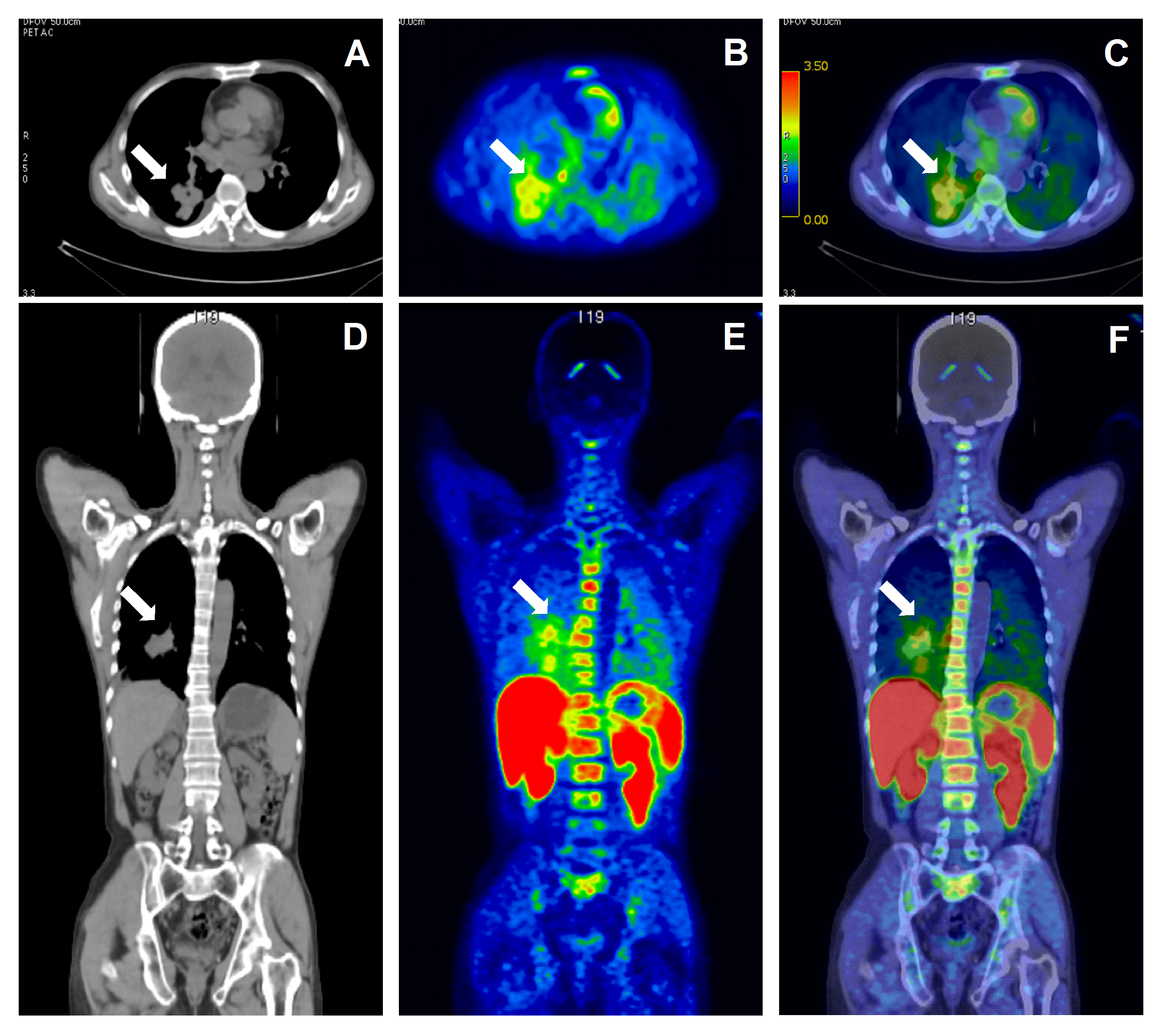


^18^F-IRS PET/CT scan in one patient with NSCLC of right lung. ( white arrow indicates tumor area) (A) Transaxial CT image. (B)Transaxial PET image. (C) Transaxial PET/ CT image. (D) Coronal CT image. (E) Coronal PET image. (F) Coronal PET/CT image.

**Fig.S3
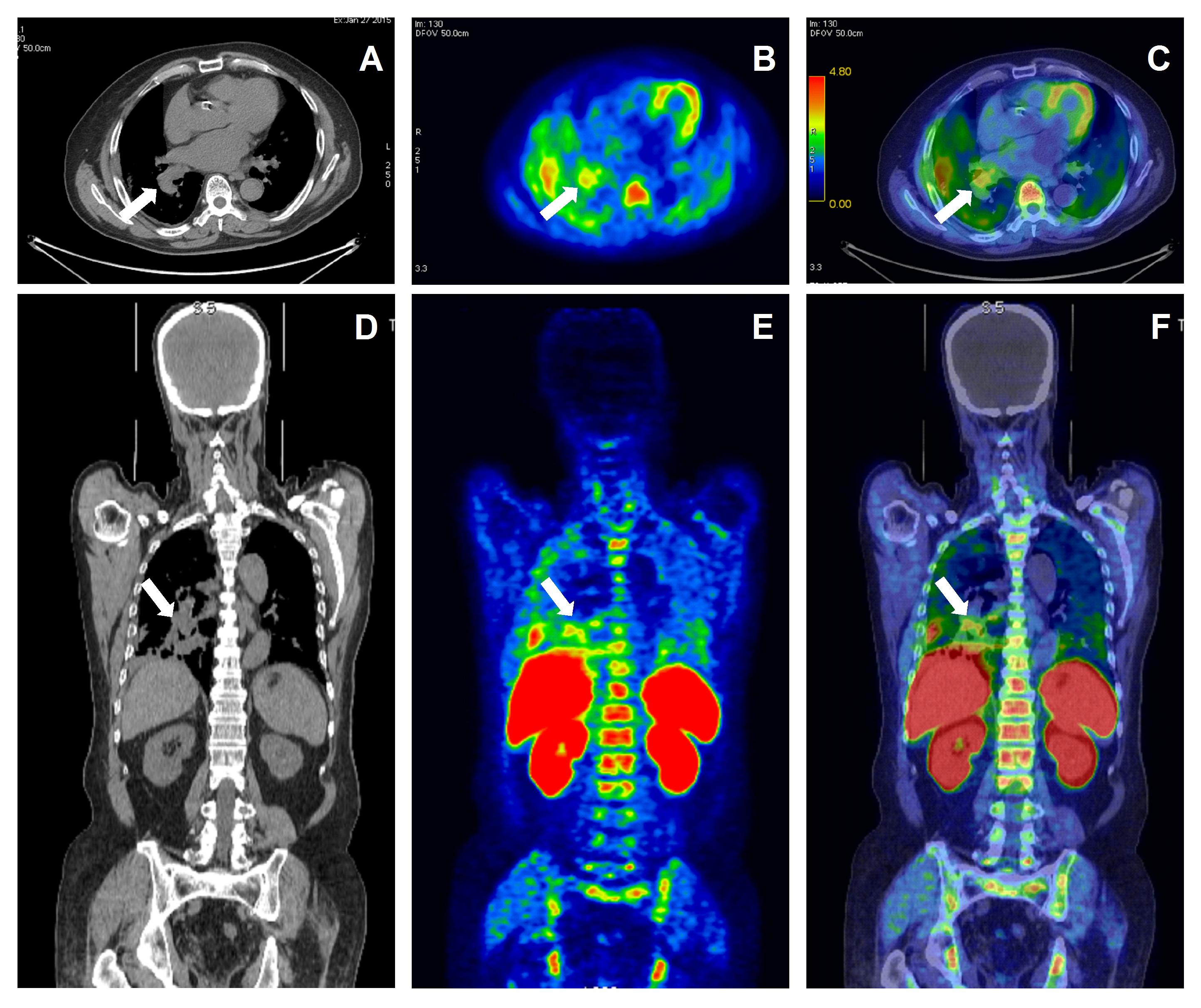
**

^18^F-IRS PET/CT scan in one patient with NSCLC of right lung. ( white arrow indicates tumor area) (A) Transaxial CT image. (B)Transaxial PET image. (C) Transaxial PET/ CT image. (D) Coronal CT image. (E) Coronal PET image. (F) Coronal PET/CT image.
